# Primary Desmoid Tumor of the Small Bowel: A Case Report and Literature Review

**DOI:** 10.7759/cureus.4915

**Published:** 2019-06-17

**Authors:** Peter A Ebeling, Tristan Fun, Katherine Beale, Robert Cromer, Jason W Kempenich

**Affiliations:** 1 Surgery, University of Texas Health Science Center at San Antonio, San Antonio, USA; 2 Surgery, Keesler U.S. Air Force Medical Center, Biloxi, USA

**Keywords:** desmoid, small bowel, resection, aggressive fibromatosis

## Abstract

Desmoid tumors, also known as aggressive fibromatosis, are fibromuscular neoplasms that arise from mesenchymal cell lines. They may occur in almost all soft tissue compartments. Primary desmoids of the small bowel are rare but potentially serious tumors presenting unique challenges to the general surgeon. We present one case of a 59-year-old man presenting with three months of abdominal distension secondary to a small bowel desmoid. Computed tomography of the abdomen showed an 18-cm mass in the mid-abdomen without obvious vital structure encasement. Percutaneous biopsy of the mass indicated a desmoid tumor. The patient underwent a successful elective exploratory laparotomy with resection and primary enteric anastomosis. Final pathology revealed the mass to be a primary desmoid of the small bowel. His post-operative course was uneventful. At two years after surgery, he is symptom free, and there is no evidence of disease recurrence. Due to the rare nature of primary small bowel desmoids, there are few specific care pathways outlined. This is a challenging pathology to treat that often requires a multidisciplinary team of surgical and medical oncologists.

## Introduction

Desmoid tumors, also known as aggressive fibromatosis, are fibromuscular neoplasms that arise from mesenchymal cell lines. Considered benign and generally not known to metastasize, desmoid tumors may be locally aggressive and cause organ compromise by mass effect. Desmoid tumors can be extra-abdominal, abdominal, or intra-abdominal [1]. In the case of intra-abdominal desmoids, the most common sites of origin are the mesentery, retroperitoneum, and small bowel. Desmoids are the most common primary tumor of the mesentery and may have a different clinical course compared to small bowel desmoids. Indeed, small bowel desmoids are exceedingly rare and offer unique management challenges to physicians. Here, we present an unusual case of one patient who underwent a successful elective small bowel desmoid resection and a review of the relevant literature.

## Case presentation

In 2013, a 59-year-old man presented to the general surgery clinic with a three-month history of gradually increasing abdominal distension associated with urinary urgency and right lower quadrant pain. He denied any weight loss, nausea, vomiting, or changes in bowel movements. Past medical history was significant for arthritis, hypertension, hypercholesterolemia, an appendectomy and colonoscopy with polypectomy in 2009. He reported no personal or family history of cancer or inflammatory bowel disease. His social history was unremarkable.

**Figure 1:**
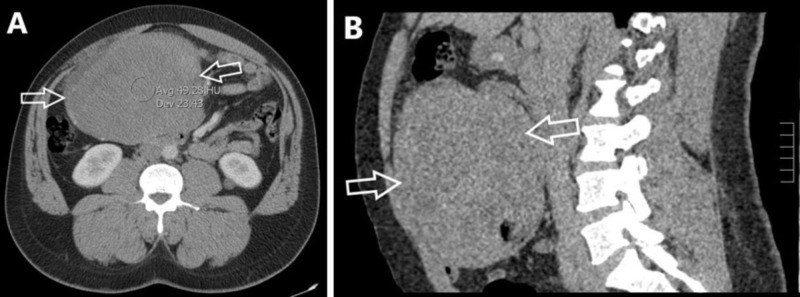
CT imaging with intravenous contrast of the abdomen and pelvis. Axial (A) and sagittal (B) CT imaging planes showing a large intra-abdominal mass (white arrows).

On physical exam, his abdomen was distended with a firm, non-tender and palpable midline mass. Computed tomography (CT) of the abdomen and pelvis with intravenous contrast revealed an 18-cm solid mass in the mid abdomen (Figure 1). The mass surrounded a loop of small bowel, but there was no radiographic evidence of bowel obstruction, bowel wall thickening, or inflammatory changes. Peripheral vascularity was present, which could represent displaced mesenteric vessels, but no communication with major vessels was noted. The mass displaced the bowel but did not appear to invade adjacent structures. Percutaneous biopsy was performed, and initial pathology indicated the mass was a desmoid tumor.

**Figure 2:**
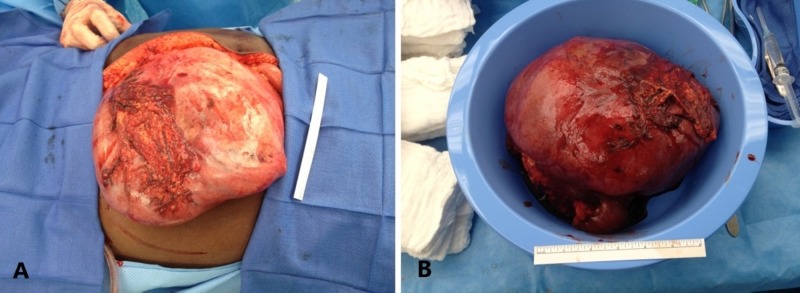
Gross pictures of the small bowel desmoid. The small bowel desmoid is plainly visible in the surgical field (A) and after resection (B).

The patient consented to an exploratory laparotomy and mass resection. The operation was carried out through a standard midline laparotomy. The mass appeared to originate from the bowel in the mid-jejunum (Figure 2). A segmental enterectomy with primary stapled anastomosis was performed.

**Figure 3:**
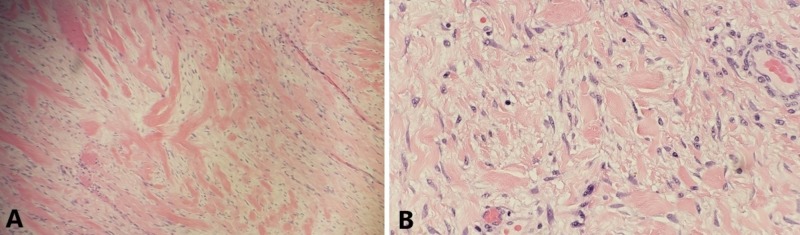
Histopathologic examination of the small bowel desmoid. Histopathology slides (A, B) stained with hematoxylin and eosin showing dense stroma with scattered cells.

Final pathology revealed a desmoid tumor originating from the small bowel with negative margins. Histopathology slides of the desmoid tumor are shown in Figure 3. The patient’s post-operative course was uncomplicated. He returned to clinic every six months for follow-up and repeat CT imaging. Two years after surgery, there was no evidence of disease recurrence.

## Discussion

Desmoid tumors are uncommon. With an incidence of two to four per million per year in the general population, they represent 0.03% of all tumors [1,2]. The histologic hallmark is spindle cells and fibroblasts in the background of a collagen stroma [2]. There are well known associations with familial adenomatous polyposis (FAP), Gardner syndrome, mutations in the adenomatous polyposis coli (APC) gene, and mutations in beta-catenin genes. It is thought that loss of function mutations in the APC gene leads to a proliferation of beta-catenin, which creates favorable environments for musculoaponeurotic growth. Other risk factors include abdominal trauma and high estrogen states. Prior abdominal surgery is also a risk factor and appeared to be our patient’s only risk factor for this pathology. While desmoids are the most common primary tumor of the mesentery, primary small bowel desmoids are exceedingly rare. There are also scattered reports of desmoids arising from the pancreas, diaphragm and gastro-esophageal junction [3-5].

There is a limited number of reports on desmoid tumors originating from the small bowel or fistulizing to the bowel [6-9]. Singh et al. report a case of a 44-year-old man presenting with a similar history and physical as our patient who underwent a segmental enterectomy for a large desmoid arising from the ileum [10]. Follow-up information is not provided. Intestinal desmoids can precipitate surgical emergencies if bowel obstruction or perforation occurs. Chang et al. describe a 50-year-old man who presented with acute peritonitis and hemodynamic instability ultimately found to have a large mass arising from the anti-mesenteric border of the ileum [7]. He did well following surgery and was disease-free at two years.

It is unknown if inflammatory bowel disease is a risk factor for the development of small bowel desmoids. However, there is increasing awareness that mesenteric desmoids may be associated with Crohn’s disease. There are isolated case reports over the past 30 years of adults with mesenteric fibromatosis who had other risk factors for desmoid development in addition to their Crohn’s disease [11-13]. Additionally, there is one report of a man with Crohn’s disease but no other identifiable risk factors for mesenteric desmoids [14]. The association has also been observed in adolescents, again, where no other obvious risk factors were present [15]. It is ultimately unclear how Crohn’s disease influences mesenteric desmoid pathogenesis. One thought is higher circulating levels of the cytokine transforming growth factor beta (TGF-β) within the intestinal mucosa may lead to enhanced fibrosis and neoplastic growth in surrounding tissues.

Practitioners should distinguish between small bowel and mesenteric desmoids, the latter being the more common intra-abdominal pathology. It is difficult to provide prognoses for either of these entities, in large part because of their rarity. One study including 52 patients with mesenteric fibromatosis at a large referral center does provide some data on disease recurrence [16]. The patient population was heterogeneous, including patients with sporadic and FAP-associated disease, as well as local and infiltrative tumors. Patients received either surgery as their first treatment, surgery and chemotherapy, chemotherapy, or observation. Of the 34 patients who received surgery as first treatment, 17.6% had evidence of disease progression up to 25 months post-operatively. However, the proportion of disease recurrence or radiographic disease instability was much lower for the entire cohort (4%) at a median follow-up time of 31.3 months. In contrast, we are not aware of similar studies pertaining to sporadic primary small bowel desmoids. It is notable that the patient we describe and at least one other in the literature were disease-free up to two years after resection [7]. This would suggest the primary small bowel desmoid may be a distinct pathologic process apart from the more common mesenteric desmoid.

**Figure 4:**
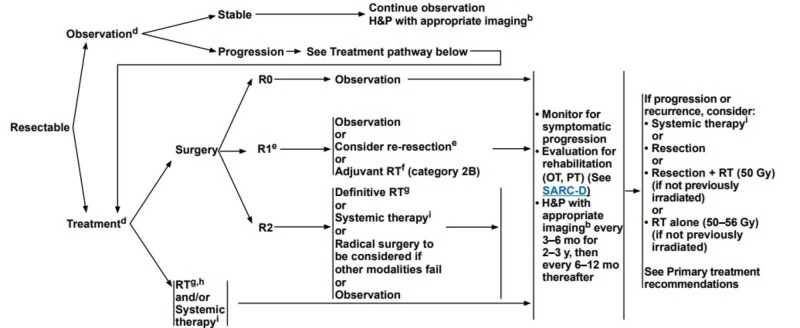
NCCN flow chart for treating patients with resectable desmoid tumors. Reproduced with permission from [17] © 2019 National Comprehensive Cancer Network, Inc. All rights reserved. The NCCN Guidelines® and illustrations herein may not be reproduced in any form for any purpose without the express written permission of NCCN. NCCN: National Comprehensive Cancer Network; R0: Microscopically negative resection margins; R1: Microscopically positive resection margins; R2: Grossly positive resection margins; RT: Radiation therapy; OT: Occupational therapy; PT: Physical therapy; H&P: History and physical; mo: Months; y: Years.

Patients with intra-abdominal desmoids would most likely benefit from multidisciplinary care from surgeons, medical oncologists, and radiation oncologists. Standardized treatment protocols for small bowel desmoids are not widely available. The National Comprehensive Cancer Network (NCCN) has outlined recommendations for the management of desmoids, although the recommendations are not specific to primary desmoid tumors of the small bowel [17]. The recommended care pathway is shown in Figure 4. In an elective setting, a biopsy should be obtained to confirm pathology. Next, the surgeon should discern between resectable and unresectable cases, or cases where surgery would be unacceptably morbid. In surgical candidates, the goal should be to achieve an R0 resection. The patient should return to clinic every three to six months post-operatively with either CT or magnetic resonance imaging (MRI) results for the first two to three years, and then follow up every six to 12 months with imaging of the relevant anatomy thereafter.

Radiation and systemic therapy are options for patients with unresectable disease, or in the setting of disease recurrence or progression. A review of 22 articles addressing intra- and extra-abdominal desmoids by Nuyttens et al. showed that surgery followed by radiation therapy or radiation therapy alone resulted in better local control than surgery alone [18]. However, the report excluded articles where patients were treated with chemotherapy. Radiation therapy is usually indicated for large, unresectable tumors and positive margins but is primarily used less for intra-abdominal desmoids due to toxicity to surrounding structures. While there are many options for systemic therapy, including nonsteroidal anti-inflammatory drugs, anti-hormonal therapy, tyrosine kinase inhibitors, and chemotherapy, the response rates are generally low [19]. Systemic therapy is an option in patients who cannot be managed by other treatments.

## Conclusions

Primary desmoid tumors of the small bowel may pose significant challenges to the general surgeon. Long-term recurrence for primary small bowel desmoids remains unclear. Elective surgery as patient condition allows is preferred, and the goal should be to achieve an R0 resection. These patients would likely benefit from having a multidisciplinary care team for treatment and monitoring. This case highlights a rare, treatable disease and the complexities of caring for patients with small bowel desmoids.
